# Rationales for Antibiotic Treatment Versus Direct Surgical Resection in Various Strata of Diabetic Foot Osteomyelitis—Literature Review and Expert Opinions

**DOI:** 10.3390/antibiotics15080797

**Published:** 2026-08-17

**Authors:** Isabel Gloor, Senta Faulhaber, Madlaina Schöni, Felix W. A. Waibel, Javier Aragón-Sánchez, Benjamin A. Lipsky, Mazda Farshad, Ilker Uçkay

**Affiliations:** 1 Diabetic Foot Unity, Department of Orthopedic Surgery, Balgrist University Hospital, University of Zurich, 8008 Zurich, Switzerland; i.gloor.94@googlemail.com (I.G.); madlaina.schoeni@balgrist.ch (M.S.); felix.waibel@balgrist.ch (F.W.A.W.); 2 Orthopedic Surgery, Limmattal Hospital, 8952 Schlieren, Switzerland; senta-faulhaber@spital-limmattal.ch; 3 Department of Surgery and Diabetic Foot Unit, La Paloma Hospital, 35005 Las Palmas de Gran Canaria, Spain; drjaviaragon@hotmail.com; 4 Department of Medicine, University of Washington, Seattle, WA 98195, USA; dblipsky@hotmail.com; 5 Medical Direction, Department of Orthopedic Surgery, Balgrist University Hospital, University of Zurich, 8008 Zurich, Switzerland; mazda.farshad@balgrist.ch; 6 Infectiology, Balgrist University Hospital, University of Zurich, 8008 Zurich, Switzerland; 7 Unit for Clinical and Applied Research, Balgrist University Hospital, University of Zurich, 8008 Zurich, Switzerland

**Keywords:** rational use of antibiotics, diabetic foot osteomyelitis, surgical resection, antibiotic treatment, rational decision

## Abstract

**Background/Objectives**: When presented with chronic osteomyelitis of the adult diabetic foot (DFO), clinicians, patients and their families have two options: rational use of antibiotics or direct surgery. **Methods**: We conducted a scientific literature review of 118 different articles and administered questionnaires to eighty DFO international and Swiss experts who have already published on the specific choice between a first-line conservative, antibiotic-based therapy versus direct surgery for DFO. **Results**: According to this specific literature and the ranking of clinical importance, the presence of ischemia came first (91% consensus favoring surgery), followed by the presence of gangrene (94% consensus), the perceived frailty of the patient (80%), sepsis (83%), and major soft tissue loss (81% consensus). Generally, for more than 90% of all experts, gangrene, bone exposed to air, destroyed bone, and hindfoot DFO motivated for direct surgery. We obtained twenty-four questionnaires from colleagues who we addressed as experts. Their opinions aligned with the literature. Compared to the international experts, Swiss clinicians were less hesitant to amputate in case of long-lasting foot ulcers, soft tissue loss, sepsis or patients with a history of low compliance. Combining literature reviews and questionnaires, large necrotic areas and destructed bone may predict direct surgery. Alternatively, clinicians can choose a first-line antibiotic therapy with minimal soft tissue debridement, off-loading and professional wound care. **Conclusions**: There is no universal consensus. The decision between antibiotics and surgery remains individualized, while severe ischemia, destroyed bone and large tissue loss are predictors of direct surgery; their absence favors antibiotic treatment.

## 1. Introduction

The optimal first-line therapy for (chronic) diabetic foot osteomyelitis (DFO) is one of the most debated issues in the treatment of new foot syndromes [1,2]. The rationales for first-line systemic antibiotic use or direct, first-line surgical resection are complex and highly influenced by the local medical or surgical culture and tradition. In the last two decades, many research groups have advocated for strict, conservative antibiotic therapy for selected DFO cases, with or without minimal debridement, to halt infection and bone destruction [3,4]. Indeed, systemic antibiotic treatment, guided by microbiological bone culture results, appears to be as safe and as effective as surgery but only in select patients with uncomplicated DFO [4], e.g., when the infection is limited to the forefoot [5,6]. Furthermore, local conditions are not the only parameters determining the initial management. The reasons for choosing first-line antibiotic therapy usually extend beyond objective decisions to include the patient’s compliance, preferences, living conditions, nutritional status, walking ability and expectations, as well as familial opinions, religious beliefs and soft tissue compromise. The surgeons’ experience and preferences, hospital policy, financial burdens, reimbursement policies, availability of a (timely) revascularization facility, local skin perfusion, lack of plastic surgery for coverage, and advanced destruction of the foot may equally play a decisive role in the complicated decision [7]. Not all of these additional rational arguments in favor of antibiotic use are, per se, evidence-based in terms of scientific evaluation.

In the long term, amputations may predispose the patient to new ulceration and infection by shifting their weight-bearing mechanics, putting new areas of the foot at risk [1,7,8,9], e.g., in the case of progressive ischemia or postsurgical wound dehiscence [9,10] after minor amputations. International guidance, which is sufficiently evidence-based regarding antibiotic regimens, cannot offer a firm recommendation for the individual decision between a first-line conservative or direct surgical approach [11]. In resource-rich settings, physicians and patients can try the conservative approach first, in combination with professional wound care, revascularization and off-loading, and switch to surgery if necessary. However, this lack of emergency, which is frequently mingled with wishful thinking, may convince doubtful clinicians to first “try conservatively”. However, the downside of this sequential approach is the need for iterative wound debridement, prolonged outpatient visits and transport, increased healthcare costs, possible selection for (Gram-negative) multidrug-resistant germs [12] and a substantial risk of antibiotic-related adverse events in 5–15% of cases [13], followed by resorting to surgery in case of (anticipated) failure.

We performed a literature review of studies in favor of first-line and rational antibiotic treatment versus surgical amputation for DFO and surveyed select experts in Switzerland and internationally. Our study concept is part of a doctoral thesis, embedded in a quality-of-care project aiming to streamline the decision-making process for the first-line approach to treating DFO in elderly patients. Of note, we do not address the (comparative) outcomes between a first-line surgical resection and the first-line antibiotic treatment for DFO, for which a much broader and evolving literature is available.

## 2. Results

### 2.1. Literature Review

I.G., I.U. and S.F. reviewed the literature published until 31 August 2023 (it is summarized in Table 1 (short version) and detailed in Supplementary Materials S1 (complete version)). In total, this review of the scientific literature yielded 118 papers reporting original data (Supplementary Materials S1). Most of the scientific literature comes from resource-rich countries in North America (*n* = 39; 33%), Europe (*n* = 67; 57%), or Asia (*n* = 9/118; 8%). These publications frequently employed an international collaboration of authors. We attributed 32 articles to the Mediterranean region (27%) and ten to resource-poor countries. Almost all were published in the 21st century in surgical (34%) or general medical (65%) (infectiology or endocrinology) journals. The surgical publications mostly originated from Spain (*Int J Low Extrem Wounds*) or the US (*Foot Ankle Surg* or *Foot Ankle Int*), probably because of the preferences of the (inter)-national opinion leaders. The specialization of the journal did not exclude mixed authorship in most papers. The opinion leader was the last author or was positioned ahead of the last author. Table 1 shows the ranking of the different clinical parameters in favor of a primarily surgical or medical approach.

The expert groups produced no clear unanimous recommendations. Numerically, there were more recommendations for direct amputation (73/118; 62%) than for the conservative approach (45/118; 38%), but these recommendations were strongly dependent on a panoply of conditions that were analyzed differently in the available literature. Surgery was very presumably also the initial purpose of many of these studies, although the conservative option was regularly acknowledged as a possible and valuable alternative. The most frequently discussed variables for the decision between surgery and antibiotic treatment were ischemia, foot architecture alterations, destroyed bone, the severity of accompanying soft tissue infection, gangrene, ulcer size, exposed bone, frailty of the patients, and, often, a history of prior amputation(s) (Table 1, Supplementary Materials S1). Among them, frequently cited parameters in favor of the direct surgical approach were severe ischemia, gangrene, sepsis, major soft tissue losses, or metatarsal head DFO [14]. In contrast, many groups advocated a conservative approach in the case of forefoot osteitis without exposed bone. Interestingly, we could not determine the maximal extent of bone destruction (cortical lysis or bone loss, sequestrum) or the extent of (lockable) skin breakdown to “allow” a conservative treatment. Surprisingly, the pathogens (e.g., fungi, multi-resistant microorganisms) or an enhanced immune suppression beyond the diabetes itself (e.g., concomitant dialysis, alcoholism, organ transplantation, immune-suppressive drugs) remained unmentioned as a decisive role.

The literature also provided (occasional) variables that were often mentioned in the papers, but astonishingly, they very rarely served as a decisional parameter regarding our study question: renal dialysis (mentioned in 26% (31/118) of the papers), neuropathy (12%), Charcot foot (3%), and large soft tissue abscess (30%). The analysis of these less frequent and “background” variables is prone to major biases due to selective discussion in a few publications and lack of own data underlying the author’s opinions. However, when dissecting their perceived importance in the few publications available, opinions in favor of surgery shifted to 94% for dialysis patients, 50% in severe neuropathy, 40% for (anticipated) drug interactions between systemic antibiotics and co-medication, and 91% for large soft tissue abscesses due to tissue loss after drainage (Supplementary Materials S1) and surgery already taking place for abscesses.

### 2.2. Online Questionnaires Administered via REDCap^™^

Following the literature review, we surveyed 24 arbitrarily selected experts for their professional opinion (via the online software REDCap^™^, version 17.2.1. Nashville, TN 37203, USA). Supplementary Materials S2 is the full-scale original questionnaire. The expert group comprised ten active infectious disease physicians and fourteen orthopedic surgeons with clinical and published academic experience in DFO management, with whom we have collaborated in the past. Three (13%) were women. The foreign experts were older than their Swiss counterparts. Among 80 experts contacted, 21 (26%) responded after the first invite in August 2023, and 3 responded after the second invite in November 2023. We grouped the experts according to whether they worked in Switzerland or abroad to detect particularities and potential biases outside and within Switzerland. The answers were complete in over 90% of the questionnaires returned. The experts took approximately 10 to 15 min to complete the survey according to our verification with five colleagues.

### 2.3. Summary of the External Experts’ Opinions, Including Figures

**Figure 1:**
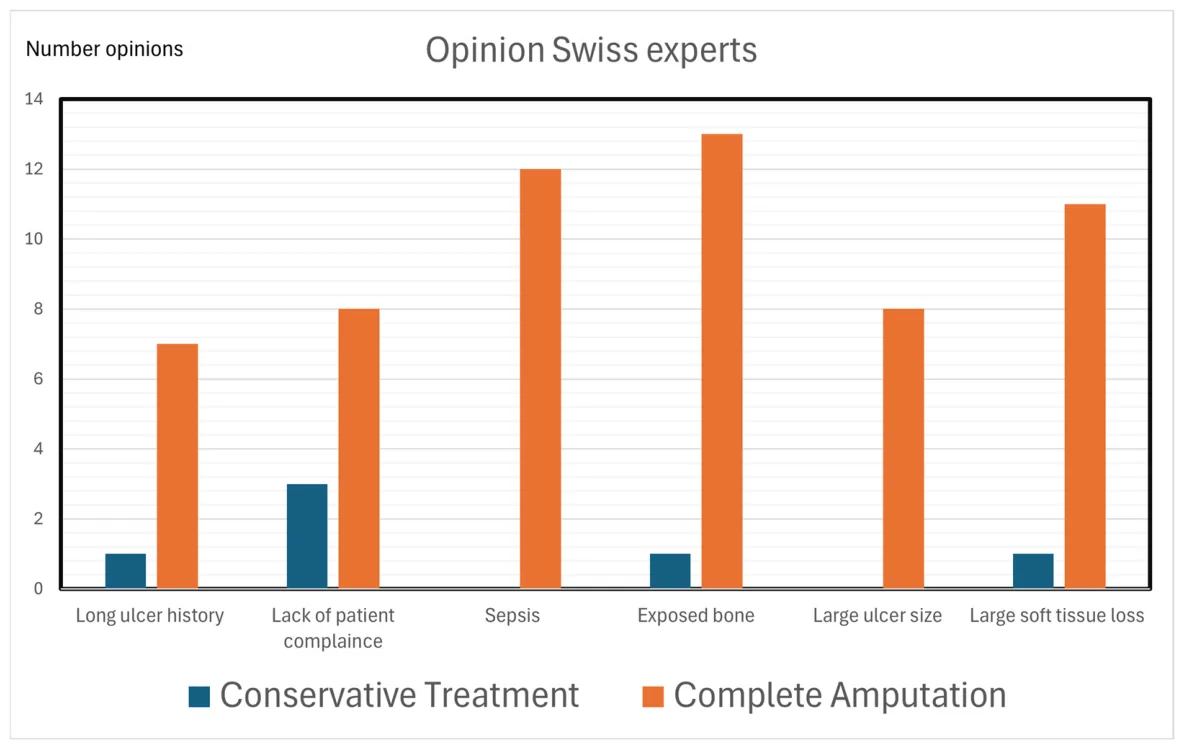
Decisional variables for Swiss experts.

**Figure 2:**
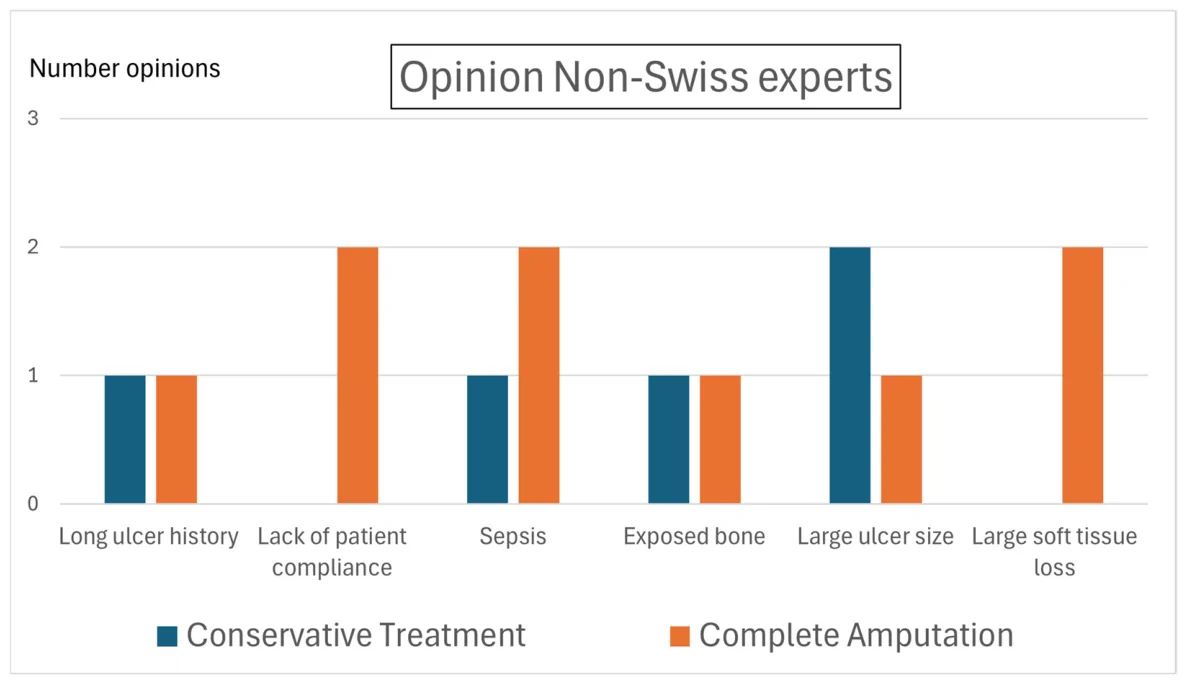
Decisional variables for international (non-Swiss) experts.

In summary, the experts’ opinions aligned with the literature (to which they themselves have contributed considerably). The general consensus was that the conservative approach can be attempted for forefoot DFO compared to hindfoot infections. Compared to the international experts, the Swiss surgeons and infectious disease physicians (and possibly the patients) were less hesitant to amputate in cases of long-lasting foot ulcers, sepsis, or patients with a history of low compliance (Figure 1). Similarly, the Swiss experts were prone to choosing first-line amputation for DFO cases with major tissue loss and consecutive difficulties with primary closure. In contrast, these soft tissue deficiencies were less important to the foreign experts (Figure 2), who probably assigned greater weight to secondary wound closure with or without plastic surgery. The opinions among the Swiss experts were not considerably different, so we may have detected a possible opinion bias among Swiss experts.

### 2.4. Patients’ Wishes

In the Swiss experts’ opinion, the patient’s preference is an important factor in the initial therapeutic decision, with a prevalence of 93.75%, and for 68.25% of experts, the wishes of the patient’s relatives would influence the decision. Among the international specialists, 100% would follow the patient’s wishes, and the preferences of the patient’s relatives and the referring physician would also influence the decision for 80% and 60% of experts, respectively. We ignore the impact of patients’ and families’ preferences after the failure of the first-line approach, as this was not part of our study questions. For the professionals, the failure of a strict first-line conservative approach often led to an ultimate surgical solution.

### 2.5. Factors in Internist’s Decision

Multi-morbidity and frailty “predict” a conservative approach, according to the opinions of 505 experts and 80% of international experts. In Switzerland, experts favor direct resection in case of the presence of local ischemia (or necrosis). In contrast, according to most foreign experts, dry ischemia predicts the conservative approach. For both national and international experts, advanced peripheral neuropathy has no influence on the treatment decision (56.25% vs. 80% in favor of surgery). Renal failure and/or dialysis have no influence on the decision in Switzerland, while foreign experts are divided fifty–fifty on this matter.

### 2.6. Surgical Factors in Decision

Previous (toe) amputation has no influence on Swiss experts’ decisions (62.5%). Internationally, such a past history is a significant element predisposed to 80% of experts choosing the conservative approach. In both groups, the presence of soft tissue abscesses favors medical treatment with bone-sparing surgery (56.25% Swiss vs. 60% international). The presence of cellulitis or erysipelas with underlying DFO does not influence the decision (31.25% national vs. 40% international, respectively) in favor of amputation. In the presence of biomechanical (architectural) disturbances, both the Swiss and international experts recommend limb-preserving surgery and, if not possible, amputation.

The most striking differences are for recommendations in cases of sepsis sensu stricto and exposed bone. Sepsis frequently triggers amputation in Switzerland, even if the patient has been stabilized. Foreign experts, especially ID physicians, are less categoric (Figure 2). In the presence of sepsis, 75% of the national specialists would favor primary amputation, while the remaining 25% would prefer preserving surgery that is limited to the (iterative) debridement of soft tissues. Internationally, 40% would opt for direct amputation. However, 20% of foreign specialists would prefer a first-line medical approach in the absence of rapidly spreading soft tissue infections such as necrotizing fasciitis or (gas) gangrene. Likewise, the majority of Swiss specialists, 75%, prefer primary amputation/bone resection in the presence of gangrene (infected necrosis). Among international specialists, 60% would perform limb-/bone-sparing surgery, while the other 40% would favor primary amputation/bone resection, like the Swiss experts.

### 2.7. Presence of Exposed Bone

While, in most studies, exposed bone predicts the direct surgical approach, the international experts surveyed are more reluctant to choose this option. Swiss specialists favor amputation, with a majority of 81.25%, in case of exposed bone, while only 20% of international experts would amputate. Among them, 60% would prefer limb-sparing or bone-sparing surgery. Similarly, among the national experts, a positive probe-to-bone test predisposes 62.5% to choose amputation, independent of the extent of bone destruction or lysis. Among the international experts, 40% prefer the conservative approach when the underlying bone is viable, 20% tend toward amputation or limb/bone-sparing surgery, and for the remaining 20%, it does not influence their treatment decision. In the presence of considerable/progredient soft tissue loss (not necessarily with exposed bone), both the Swiss and foreign experts would opt for direct resection (75% vs. 60%, respectively).

### 2.8. Other Aspects of the Decision

The experts also spontaneously mentioned other aspects we did not specifically ask about. Internationally, the availability of skilled and experienced surgeons, as well as rehabilitation facilities, would influence the decision between the two initial approaches. The Swiss participants repeatedly emphasized the importance of an interdisciplinary conference or any multidisciplinary decision-making processes. Another element is vascular disease. According to many experts, clinicians should address vascular pathologies before scheduled surgery. If there is a striking improvement after reperfusion, then bone-sparing surgery, as a second-line decision, is preferred.

## 3. Discussion

DFO is difficult to treat, with two major first line therapeutic approaches (antibiotic treatment versus resection). The rationale for each individual decision is usually complex, multidisciplinary, and multifaceted. Indeed, the final outcome can be very different depending on several patient-, wound- and infection-specific factors [15]. DFO management usually requires a multidisciplinary approach involving a wide variety of medical, surgical and other healthcare professionals, as well as patient compliance [1,8]. Guidelines from the Infectious Disease Society of America (IDSA) [1] and the International Working Group on the Diabetic Foot (IWGDF) [11] provide many evidence-based recommendations for multiple aspects of DFO. However, they do not provide in-depth guidance regarding the first-line approach. The updated IWGDF/IDSA guidelines from 2023 recommend considering antibiotic treatment without surgery in cases of uncomplicated forefoot DFO [11]. However, in cases involving increasing ulcer sizes, higher wound grades and arterial calcifications in the foot, the literature points to a surgical approach [7,16,17,18,19,20,21,22].

As updated guidelines on this particular topic have not been issued in Switzerland, we conducted a narrative literature review on the most critical factors determining the choice of a conservative or surgical approach to DFO. Based on these results, we prepared a questionnaire that was administered to leading experts in the fields of infectiology and orthopedic foot surgery. Aside from the patient’s preference, the three most influential variables favoring first-line surgical amputation are exposed bone, large areas of soft tissue loss (that cannot be closed by flap) and the presence of gangrene (Table 1). The only factor clearly favoring a conservative approach is forefoot (toe) DFO. Faglia et al. state that “A higher rate of transtibial amputation is found when osteomyelitis involved the heel instead of the midfoot or forefoot in diabetic patients” [16], which has been confirmed by other groups [18]. The same is true of sesamoid osteitis, which frequently recurs if not surgically resected [19,20].

Another important aspect is peripheral vascular (arterial) disease. If successful revascularization is improbable, many surgeons refrain from amputation. This decision may also consider transcutaneous oxygen pressure, a semi-objective factor, although its predictive value for uneventful wound healing is biased by many skin conditions, such as edema, and lacks strong scientific evidence in terms of predicting success [23]. In the landmark paper by Tone et al. on the duration of systemic antibiotic administration for conservatively treated DFO, patients were excluded in the case of absent anterior and posterior pedal pulses during Doppler arterial examination [24]. Notably, the experts we interviewed did not spontaneously emphasize these detailed parameters, as we did not specifically ask them about vascular aspects. Other variables seem less important. Indeed, there are many important parameters in treating DFO that do not influence the decision between conservative treatment and direct resection. These include microorganisms present in the bone, the antibiotic resistance of the pathogens, the duration of antibiotic administration, the cost of appropriate antibiotic agents, the size and duration of the underlying ulcer, the degree of polyneuropathy and laboratory results [25].

In a specific opinion paper, Prof. Lipsky proposed six (surgical) aspects in favor of a conservative approach “patient is too medically unstable for surgery; poor postoperative mechanics of foot is likely (e.g., with mid- or hindfoot infections); no other surgical procedures on foot are needed; infection is confined to small, forefoot lesion; no adequately skilled surgeon is available; surgery costs are prohibitive for the patient; patient has strong preference to avoid surgery) and another six against (foot infection is associated with substantial bone necrosis, foot appears to be functionally non-salvageable, and patient was already non-ambulatory; patient is at particularly high risk for antibiotic-related problems; infecting pathogen is resistant to available antibiotics; limb has uncorrectable ischemia (precluding systemic antibiotic delivery); patient has strong preference for surgical treatment”. These latter recommendations are obviously ID-based. While they perfectly encompass the antibiotic aspects, they may not be fully relevant to the surgical clinical mindset [26].

In 2019, another expert group led by Prof. Lázaro Martínez (podiatry, surgery) published twelve (other) criteria for the medical approach (there is no persisting sepsis associated with DFO. Patients can receive and tolerate appropriate antibiotic therapy. The degree of bone destruction has not caused irretrievable compromise to foot mechanics. The patient prefers to avoid surgery. The patient’s comorbidities confer high risk to surgery. There are no contraindications to prolonged antibiotic therapy. Surgery is not otherwise required in adjacent soft tissue infection or necrosis. Infection is confined to small forefoot lesions that are easily off-loaded. Patients have good vascular status that allows drug spreading and tissue availability. No adequately skilled surgeon is available. Operating room and other surgical facilities are not available. Surgery cost prohibits the patient from undergoing the surgery) and five favoring surgeries: DFO with systemic toxicity associated with soft tissue infection; substantial cortical destruction, osteolysis, macroscopic bone fragmentation (sequestration), or necrotic bone seen on X-ray; visible, chronically exposed trabecular bone identified within a forefoot ulcer; open or infected joint space and prosthetic heart valves) [27]. The number of recommendations is too high, but the review also mentions a widespread belief that open toe articulations must be resected and that patients with prosthetic heart valves (presumably also pacemakers) should be treated surgically for infection [27]. The open-joint issue was also addressed by Prof Aragón-Sánchez [20] and colleagues, who state that bony surfaces in the interphalangeal and metatarsophalangeal joints are covered with a layer of hyaline cartilage or fibrocartilage within a joint cavity that contains synovial fluid, lined with a synovial membrane and reinforced by a fibrous capsule and ligaments. To reach a joint, the infection must go through the capsule, and significant destruction of this fibrous tissue, which is poorly vascularized, is frequently observed. This means that infection may remain in this fibrous tissue if not carefully removed. Another consequence is that when the joint is involved, it should be assumed that the infection affects both bones [20].

Prof. Aragón-Sánchez also stated the following in 2015: “Surgery is required when the bone is protruding through the ulcer; there is extensive bone destruction seen on x-ray or progressive bone damage on sequential x-ray while undergoing antibiotic treatment, and the soft tissue envelope is destroyed; and there is gangrene or spreading soft tissue infection.” Moreover, it is necessary to have a surgeon with diabetic foot expertise available, because in cases in which osteomyelitis is associated with a bone deformity, surgery is an appropriate treatment [20]. This agrees with our own clinical experience. In 2021, a more rigorous systematic review examined the scientific evidence supporting medical versus surgical treatment for the management of DFO [22]. That review included six clinical trials with a total of 308 participants; the conclusions were that (a) the lack of homogeneity in the treatments applied in the studies complicated attempts to perform meta-analyses and to analyze the rates of DFO remission or complication between two types of treatments and (b) that the available evidence is insufficient to identify the best option to cure DFO [22].

**Figure 3:**
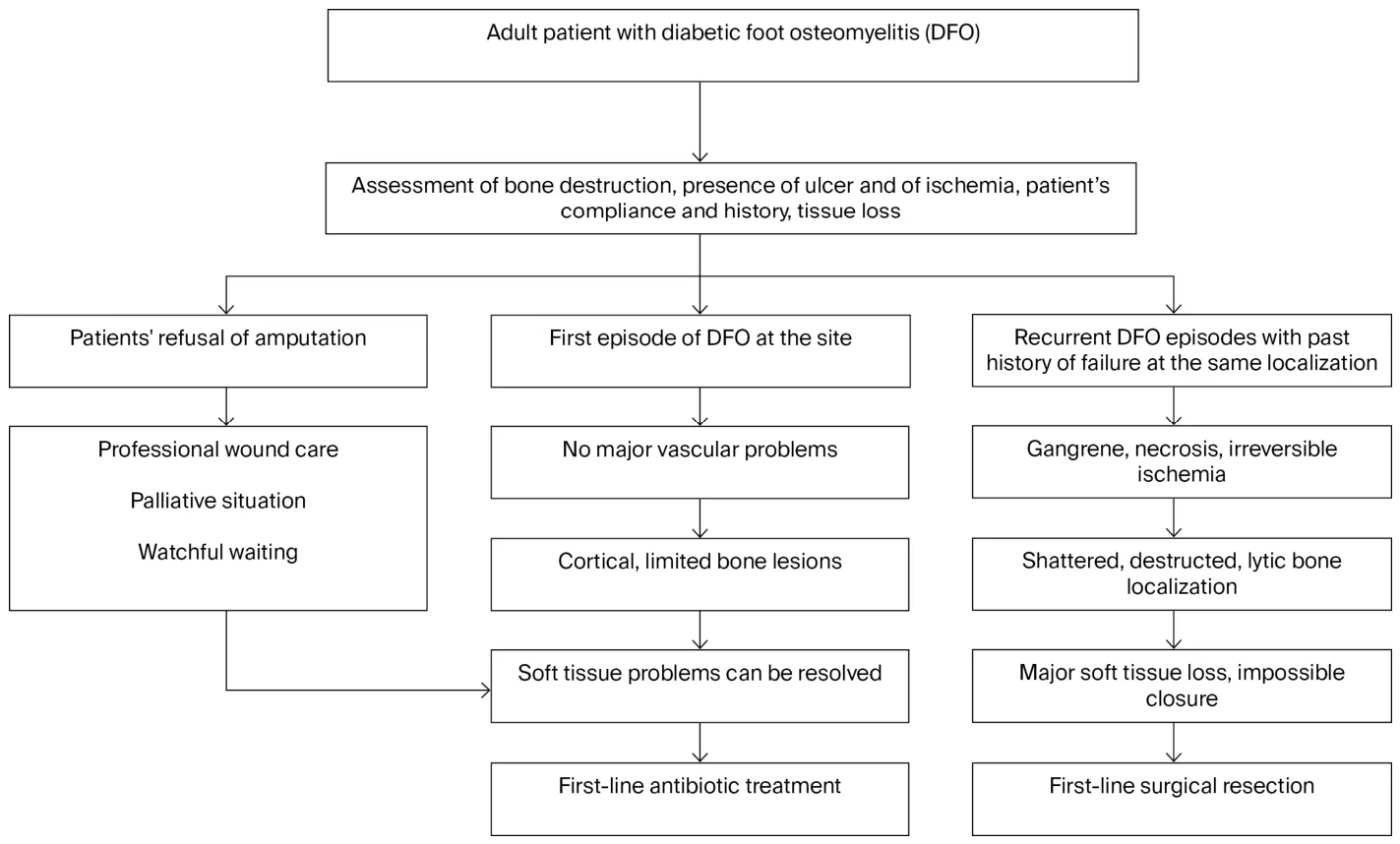
Proposition for a minimal rudimentary decisional algorithm regarding the first-line choice of DFO management.

In conclusion, it is difficult to decide between the two approaches. There is no universal consensus, and the decision between antibiotics and surgery remains individualized. However, documented terminal ischemia, a destructed bone, and chronic tissue loss are common indicators for a surgical approach, and their absence favors antibiotic treatment. Figure 3 could be such a proposition for a rudimentary decisional algorithm.

Our work has important limitations. (a) We conducted an opinion survey instead of analyzing composite original data. Likewise, our primary study question was expert opinion regarding the initial clinical decision-making and not a comparison of outcomes between a surgical versus medical approach for first-line DFO management, which is a different study question. (b) We chose a classical medical and surgical review and questionnaire design. A different design, such as that used in psychology or psychiatry, would give further weight to the certitude of an opinion. (c) We assessed the opinions of experts working in resource-rich countries and only a few of them. Accordingly, financial, political and resource-related issues were not considered in the choice of first-line approach. It is very likely that the opinions and practices of experts working in resource-poor settings would be more influenced by local reimbursement politics, resource availability and potentially greater variability in patients’ beliefs and opinions [28]. Similarly, we addressed experts who published on the decision-making between a first-line surgical resection versus an antibiotic treatment attempt in the past. Of course, there are many more experts (groups) worldwide who go unrepresented because they did not explicitly publish (in scientific English) on this specific study question, even if they treat DFO patients every single day. Globally, this “missed number of experts” is probably in the thousands. (d) Formally, we assessed opinions specifically concerning conservative treatment vs. surgical resection/amputation; as a result, we cannot comment on other techniques such as excessive curettage [29] without resection or percutaneous partial bone excision [30]. Similarly, there is no sharp and definitive distinction from evidence in the available literature, personal opinion and personal experience, not even among the authors of this manuscript let alone with other groups, across time, medical disciplines, settings or geographical regions. (e) We concentrated on the conditions present at the time of a given DFO episode. Beyond this crucial time, when a therapeutic decision is pending, there are chronic circumstances known from the patient’s past that are generally valid for medical decisions; these include the patient’s co-morbidities [31] (e.g., dementia), general adherence to drug therapies [32] and willingness to wear an uncomfortable cast after surgery [33]. Although important, the discussion of general medical compliance was beyond the scope of this manuscript. (f) We questioned a standard surgical technique (amputation, resection) and compared it with a standard conservative approach using systemic antibiotic agents. These approaches are not exclusive, as the literature provides other possibilities as well that were not assessed in our study. DFO treatment may encompass bioabsorbable and non-absorbable (antibiotic) carriers, calcium sulfate beads, hydroxyapatite ceramic, collagen sponges, bioactive glass, and immunomodulatory strategies, including mesenchymal stem cell-based therapies, cytokine-targeted interventions, bacteriophages, and quorum-sensing inhibitors, which demonstrate promising infection resolution rates and a potential to reduce the surgical burden, though most are limited by small cohorts and observational designs [34,35,36]. Similarly, alternative surgical approaches (i.e., cancelloplasty, conservative bone excision, and tibial cortex distraction) have shown early success in limb preservation. Nonoperative strategies, including antimicrobial peptides and topical oxygen, offer additional options, particularly for patients unfit for surgery [34]. Likewise, the conservative antibiotic approach is very different across the centers in a single country, let alone between countries or different settings, limiting the generalizability of the antibiotic approach [37]. (g) Finally, there is one difference between medical and surgical DFO treatment that is not important for the immediate treatment of DFO but may play a preventive role in the future. In contrast to antibiotic treatment that only addresses the infection, surgical intervention treats the current episode while preventing future problems, e.g., by performing a tenotomy on non-infected toes [30,38] or by correcting an advanced pathological foot architecture due to long-standing neuropathy [39]. Indeed, this last preventive aspect, which is only effective in the future, is often neglected for first-line treatment. In the literature, current experts advocate that, despite a higher rate of rehospitalizations and a high failure rate, in patients with mild and limited DFO, it may be reasonable to offer a primary nonoperative treatment for digital osteomyelitis [40].

## 4. Materials and Methods

### 4.1. Setting

Balgrist University Hospital is a referral orthopedic center. We aim to streamline and facilitate the timely management of DFOs through multiple (inter)-national research projects. In our policlinic, we regularly discuss the best approach to treating DFO in the context of patient comorbidities and the condition of the affected foot. Although we shortened the administration of systemic antibiotics in several retrospective [41] and prospective–randomized trials [42], we are less confident about the correct indications for surgery at the first diagnosis of DFO. This research was performed as part of a doctoral thesis.

### 4.2. Literature Review

I.G., S.F. and I.U. performed an extensive (narrative) literature review by searching PubMed and Google Scholar using the MeSH terms “antibiotic”, “amputation”, “resection”, “conservative”, “diabetic foot osteomyelitis”, or “guidelines” in different combinations in German and English without time or geographical restrictions. We particularly emphasized the literature stemming from resource-poor settings, if available. We were interested in the parameters and clinical variables used for clinical decision-making between a (first-line) direct surgical resection and a (first-line) conservative antibiotic treatment of DFO in adult patients. Therefore, we excluded all papers that only reported the outcome of either approach without providing sufficient rationale for the therapy chosen. We summarized the findings in Supplementary Materials S1 by breaking the information down into 18 variables frequently encountered in the literature: renal failure, patient comorbidities, limb ischemia, peripheral neuropathy, prior amputation, abscesses, exposed bone, degree of bone destruction, ulcer size/wound grade, gangrene, necrosis, severity of soft tissue infection, soft tissue loss, systemic infection/sepsis, drug interactions and side effects of the antibiotic therapy, biomechanical foot deformities, and localization of DFO. We excluded case reports, papers reporting only a widespread opinion, and articles addressing diagnostic or epidemiological issues of DFO (Table 1, Supplementary Materials S1). This review took 180 h. We ended the search on 31 August 2023.

### 4.3. Questionnaires

Based on our own experience and the literature review, we organized relevant clinical questions into an online questionnaire using REDCap^™^ (version 17.2.1. Nashville, TN 37203, USA). Research Electronic Data Capture) software with the help of UCAR (Unit for Clinical and Applied Research). We created a German version (for domestic use) and an English version (Supplementary Materials S2). The questionnaire was composed of four direct questions (yes or no), 24 four-item scaled questions, and 1 free-text answer. Before emailing the invited experts twice in 2023, we comprehensively validated the questionnaire according to Balgrist uncertainties.

Internationally, we chose eighty experts working in infectious diseases, vascular surgery, diabetology and orthopedic foot surgery with whom we had some degree of professional and/or academic collaboration in the past and who had already published in PubMed and on the Internet (with own data) with respect to our study question, which is the specific decision-making between a first-line surgical resection versus conservative, medical antibiotic treatment for DFO. Importantly, and according to this specific study question, we did not address experts who published on the outcomes of DFO management according to the management chosen. This latter literature would have been much more abundant. Many experts were active at the 2023 International Guidelines on Diabetic Foot (IWGDF) [11] and in previous versions of international or IDSA (Infectious Diseases Society of America) [1] guidelines. This iterative international academic activity helped identify the e-mail addresses of international experts. Indeed, during the systematic review for the IWGDF guidelines, with the help of professional librarians, professional epidemiologists, and the Covidence^™^ software (Covidence systematic review software (2022), Veritas Health Innovation Ltd., Melbourne 3000, Australia), we came across the expert lists of these guideline committees. Moreover, for decades, we have been conducting international research on DFO management and know (English-speaking) colleagues all over the world. Many of us continue to review high-standing papers on DFI (including ntibiotics MDPI, for which we also served as Guest Editors on a Special Edition).

Besides our own lists of international colleagues, three authors (I.G., I.U., and B.A.L.) identified experts unknown to us via an independent internet search and controlled the number of publications of the corresponding author groups. The experts were very often the first or the last authors in an elevated number of publications. In the setup of our expert search, we purposely accepted the following biases: (a) publication bias, as we only accepted experts who have published previously on our study question; (b) seniority biases, as we only ask colleagues with a long experience in this field; (c) clinician bias, as we only target clinicians; and, most importantly, (d) response bias, as we can only consider opinions that are voluntarily returned during the few weeks of opportunity.

To recruit experts in Switzerland, we sent the REDCap^™^ link to colleagues known for their engagement in national diabetic foot problems [43]. As for the international experts, we did not contact professionals who were experts in other specific aspects of diabetic feet such as off-loading, orthoses, wound debridement, education, physical therapy, microbiology, histology [44], insurance, reimbursement, or revascularization.

### 4.4. Statistical Analyses

We used only descriptive statistical analyses.

## Figures and Tables

**Table 1:** The five most frequently expressed opinions are indicated in bold and underlined.

118 Articles	Ischemia	Gangrene, Necrosis	Frail Patient	Sepsis	Soft Tissue	Bone Destruct.	Bone Exposed	Ulcer Size	Past Resection	Infection Hindfoot
Conservative	5	3	9	7	6	1	0	1	2	1
Surgery	51	44	36	35	25	21	13	13	10	13
Ranking	1.	2.	3.	4.	5.	6.	8.	10.	10.	12.
Proportions	91%	94%	80%	83%	81%	95%	100%	93%	83%	93%

## Data Availability

Key data are available upon reasonable scientific request to the corresponding author.

## Supplementary Materials

The following supporting information can be downloaded at https://www.mdpi.com/article/10.3390/antibiotics15080797/s1. Extended summary of the literature tables [20,42,45,46,47,48,49,50,51,52,53,54,55,56,57,58,59,60,61,62,63,64,65,66,67,68,69,70,71,72,73,74,75,76,77,78,79,80,81,82,83,84,85,86,87,88,89,90,91,92,93,94,95,96,97,98,99,100,101,102,103,104,105,106,107,108,109,110,111,112,113,114,115,116,117,118,119,120,121,122,123,124,125,126,127,128,129,130,131,132,133,134,135,136,137,138,139,140] of the questionnaire. Supplementary Materials S1 provides details of our literature review. Supplementary Materials S2 is the (online) questionnaire in English language.

## Abbreviations

The following abbreviations are used in this manuscript:

DFO	Diabetic foot osteomyelitis.
