# Supplementary material for: Rationales for Antibiotic Treatment Versus Direct Surgical Resection in Various Strata of Diabetic Foot Osteomyelitis—Literature Review and Expert Opinions
Source: Antibiotics (Basel). 2026 Aug 17;15(8):797. doi: 10.3390/antibiotics15080797 (PMC13509231; doi:10.3390/antibiotics15080797)
Supplement: Supplementary file 1 [file antibiotics-15-00797-s001.zip › antibiotics-4365861-supplementary/Supplementary Materials S1.pdf]

References	Country & speciality	Treatment	Clinical Factors																
			**Renal failure**	**Limb ischemia**	**Peripheral neuropathy**	**Previous amputation**	**Abscess**	**Exposed bone**	**Ulcer size/ wound grade**	**Gangrene**	**Necrosis**	**Severity of soft tissue infection**	**Bone destruction**	**Soft tissue loss**	**Severity infection / sepsis**	**Drug interactions and side effects of the antibiotic therapy**	**Foot alterations**	**Localisation**	
																		fore foot	heel
45	Italy, Endocrinologist	conservative																	
		surgical	x	x	x	x			x	x					x				
46	Turkey, Infectiologist	conservative																	x
		surgical	x	x	x	x			x		x				x				
47	USA, Internal specialist & emergency medicine	conservative																	
		surgical	x	x															
1	USA, Infectiologist	conservative													X, non-persisting (-71h)	X, if too unstable for surgery		x	
		surgical	x	x			X	x		x	x		x	x	X, persisting /life or limb threatening	x			x
48	USA, Infectiologist	conservative																	
		surgical		x							x	X(widespread)	x				x		x
49	China (meta-analysis of English studies), Endocrinologist	conservative																	
		surgical				x				x					x				
50	France, Infectiologist	conservative																	
		surgical	x	x	x	x												x	
51	Korea, Pharmacist	conservative																	
		surgical	x	x		x			x									x	
9	USA, Infectiologist	conservative																	
		surgical		x															
52	Switzerland (review of English and French language) Infectiologist	conservative																	
		surgical		x			x				x				x				
16	Italy, Endocrinologist	conservative																	
		surgical	x			x									x				x
53	UK, Endocrinologist	conservative																	
		surgical	x	x				x								x			
54	USA, Infectiologist	conservative																	
		surgical								x	x				x	x			
6	France, Infectiologist	conservative														X, if to unstable for surgery	X, postoperative	x	
		surgical		x							x	x	x			x			
55	UK/Italy, Orthopaedic surgeon	conservative																x	
		surgical									x					x			x
56	USA/UK, Infectiologist	conservative																	
		surgical	x	x						x	x				x				
57	Netherlands, Internal specialist	conservative																	
		surgical	x																
87	USA, Infectiologist	conservative												X (if minimal)				x	
		surgical		x			x			x	x		x	X (if extensive)	x		x		x

59	UK, Orthopaedi csurgeon	conservative			x													x	
		surgical		x			x	x			x	x				x	x		
5	Spain/UK, Surgeon	conservative														X, too unstable for surgery	X (to avoid if induced by surgery	x	
		surgical	x	x			x	x		x	x	x	x	x	x	x	x		
		surgical		x				x			x	x	x		x				
22	USA/UK/Switzerland, Infectiologist	conservative														X, too unstable for surgery	X (to avoid if induced by surgery	x	
		surgical		x			x	x	x	x	x	X (spreading)	x	x	x	x	X, nonsalvageable		
60	UK/Turkey/Switzerland, Infectiologist	conservative																x	
		surgical		x			x			x	x				x				x
24	USA, Infectiologist	conservative																x	
		surgical		x			x			x	x				x	x	x		
7	USA, Infectiologist	conservative																	
		surgical																	
61	Pakistan, Surgeon	conservative																	
		surgical			x				x			x			x				
15	USA, Student/ Orthopaedi c surgeon	conservative																	
		surgical		x	x														
62	China, Endocrinologist	conservative																	
		surgical	x																
29	Spain, Podiatricsurgeon	conservative			x												X	x	
		surgical	x	x				x			x	x			x	x	x		
24	France, Infecti ologist	conservative			x									X if minim al			X i	x	
		surgical	x	x						x	x	x	x			x			
42	Germany, Orthopaedic surgeon	conservative			x														
		surgical	x	x			x	x		x	x		x	x	x				
63	Germany, Endocrinologist	conservative			x														
		surgical		x			x			x	x		x	x		x			
64	Germany	conservative																	
		surgical		x				x		x	x				x				
66	Swi tzerland, Geneva	conservative																	
		surgical														X preference			
67	Switzerland, Geneva, Infection Control	conservative					x				x	x							
		surgical	x	X (low oxygen levels)											x				
68	USA, Foot Ankle Clin	conservative										X mild/moderate		X if no sepsis					
		surgical		X (high risk amputation)			X drainage				x	X severe			x				
69	UK, Med Clin North Amp	conservative																	
		surgical																	
70	USA, Ann Surg	conservative																	
		surgical	x	x												x			
71	UK, Diabetologica	conservative													X less severe				
		surgical					x				x								
72	USA, Clinical Infectious diseases	conservatove																	
		Surgical General pro surgery					x			x									
73	USA, Podiatry, Foot Ankle Surg.	conservative				x													
		surgical				x													
74	USA, Foot Ankle Int.	conservative																	

		surgcical													x	X (Malnutrition as risk)			
75	USA, Clin POdiatr Med Surg	conservative												X early					
		surgical					x				x		x						
76	USA, American Diabetes Association	conservative														x	X (poor mechanics postop)	X)	
		surgical		x						x		x	x		x		x		
27	Spain, Podiatry	conservative													X no sepsis	X too ill	X no	X	
		surgical																	
77	Italy, Foot Ankle Surg	conservative		x															
		surgical					x						x						x
78	India, Diabetes Metab Syndr	conservative													X milde/modarate				
		surgical					x			x	x				x		x		
79	USA, Foot Ankle Surg	conservative																	
		surgical															X Charcot		
80	USA, Int J Low Extrem Wounds	conservative										X MRSA			X SIRS				
		surgical	x	x								X pseudomonas , e.coli				X low albumin, nicotin,		X	
81	China, Pak J Med Sci	conservative																	
		surgical		X			X		x	X	X	X							
8	Spain, Podiatry	conservative														x	X	x	
		surgical		X untreatable									X bone necrosis						
3	USA, mixed disciplines	conservative											X chronic OM	X less severe			X		
		surgical					X				X	X			X				
82	Turkey, Hematol Rep	conservative																	
		surgical													X higher				
83	Switzerland, Zurich	conservative																	
		surgical	X	X												X immunsuppressiva	X charcot osteomyeltisi		
84	Turkey, diabetology	conservative	x												x				
		surgical	x												x				
85	USA, Am J Med.	conservative																	
		surgical								X	X								
86	USA, Open Forum Infect. Dis.	conservative																	
		surgical	x									X Polymicrobial infection			X (low Albumin)				
87	Spain, Podiatry	conservative																x	
		surgical					x	x			x		x						
88	UK, Diabets Res Clin Pract	conservative																	
		surgical	x									X MRSA associated with treatment failure			x				
89	Argentina , Int J Low Extrem Wounds	conservative												X less severe					
		surgical	x				x				x	x		x					
90	Spain, Surgery, Canarias	conservative																	
		surgical	x								x	x							
91	Spain, Surgery, Canarias	conservative																	
		surgical													x				

92	Spain, Surgery, Canarias	conservative																	
		surgical	x					X risk amputation			x								
93	Switzerland, Zurich	conservative																	
		surgical	x									x			x				x
26	USA, Infectious Diseases	conservative														x		x	
		surgical	x										x		x				
94	Spain, Surgery, Canarias	conservative																	
		surgical																X	
95	Spain, Enferm Infecc Microbiol	conservative																x	
		surgical		x					x					y					
96	Spain, Costa Rica	conservative												x					
		surgical												x					
97	USA, Am Fam Physician	conservative												X moderate				x	
		surgical		x									x		x				
98	USA, Am J Surg	conservative																x	
		surgical		x			x				x		x						
99	USA, Foot Ankle clin	conservative																	
		surgical																	x
100	UK, Vasc Health Ris Manag	conservative												X not life threatening					
		surgical		x			x						x						
101	USA, Clin Podiatr Med Surg	conservative																x	
		surgical		x							x								
102	France, Montpellier, Nîmes	conservative																x	
		surgical	x			x			x					x					
103	Spain, Surgery, Canarias	conservative																	
		surgical		x							x	x							
104	USA, Clin Podiatr Med Surg	conservative										X mild/moderate							
		surgical		x						x	x	x	x		x				
105	United Arab Emirates	conservative																	
		surgical		x					Osteitis, high Wagner						x				
106	UK, PloS One	conservative																	
		surgical								x					x				X poot putcome
107	China, Front Endocrinol	conservative																	
		surgical		x		x					x		X OM						
108	Korea, Vascular Specialist	conservative																	

		surgical		x					X high wagner										
109	Indonesia, World J Orthop	conservative														X patient preference			
		surgical		x									X Charcot						
40	Israel, J Am Podiatr Med Assoc.	conservative													X mild/limited				
		surgical													x				
110	Spain, Surgery, Canarias	conservative																	
		surgical									x				x				
111	International Expert Group, infectiology	conservative		X forefoot without PAD			X forefoot without	X forefoot without										x	
		surgical					x								x				
112	USA, Podiatry, JAMA	conservative												X forefoot					
		surgical																	
113	USA, Infectiology	conservative																x	
		surgical		x			x			x		x							
114	France, Infectiology	conservative										X mild/moderate						X pedal	
115	UK, Diabet Med	conservative	x	X								X MRSA							
		surgical																	
116	France, Diabetes care	conservative											X if antibiotic guided						
		surgical																	
117	France, Tourcoing, Lille	conservative																	
		surgical		x			x								X high CRP				
118	Spain, Surgery, Canarias	conservative																	
		surgical													x				
119	Canada, Foot Ankle Int	conservative														X all others treated with AB			
		surgical		x			x					x							
120	Lebanon, Foot ankle Surg	conservative																	
		surgical																	X
121	Saudi Arabia, Diabetes Metab Syndrom	conservative								X dry									
		surgical					x			X wet		x			x				
122	USA, Foot Ankle Clin	conservative																	
		surgical		x	x						x	x							
123	Turkey/USA, CMAJ	conservative																x	
		surgical																X	
124	Spain, Surgery, Canarias	conservative													X mild				
		surgical					x					x			X				
125	USA, Surg Infect	conservative													X if no pseudomonas, e coli				
		surgical												X positive margin		X low albumin		x	
126	Switzerland, Zurich	conservative																	
		surgical																	
127	Iraq, Open Access Maced J Med Sci	conservative													X mild/moderate				

		surgical																	
127	Mexico, Wound Repair Regen	conservative																	
		surgical	x												X polymicrobial				
128	Sudan, Diabet Foot Ankle	conservative		X no ischemia			X no abcess				X no necrosis								
		surgical		X critical			x			x	x	x							
129	Italy, J Diabetes Complications	conservative																	
		surgical			X charcot														
130	Spain, Surgery, Canarias	conservative																	
		surgical						x					x						
20	Spain, Surgery, Canarias	conservative																	
		surgical		x		X first ray		x		x		x	x	x					
131	USA, Surgery	conservative																	
		surgical		X combined with ulcer															x
132	Sweden, Endocrine	conservative																	
		surgical																	
133	Sweden, J Diabetes Complications	conservative																	
		surgical					x			x	x				x				
18	UK, Int J Low Extrem Wounds	conservative																	
		surgical		X absence pedal pulses														X	
134	France, Diabetes Metab	conservative				X risk for reulceration													
		surgical																	
135	France, Diabetes care	conservative		X no severe	x														
		surgical																	
41	France, Int J Low Extrem Wounds	conservative																	
		surgical																	
136	Spain, Surgery, Canarias	conservative																	
		surgical																	
137	USA, Clin Infect Dis	conservative																	
		surgical																	
138	USA, Am J Med	conservative		X higher risk for failure	X higher risk for failure														
		surgical																	
139	USA, Diabetes Metab Res Rev.	conservative													X mild				
		surgical		x			x			x	x	x			x				
140	Spain, various associations	conservative																	
		surgical					x			x	x	x			x				
