# Supplementary material for: Rationales for Antibiotic Treatment Versus Direct Surgical Resection in Various Strata of Diabetic Foot Osteomyelitis—Literature Review and Expert Opinions
Source: Antibiotics (Basel). 2026 Aug 17;15(8):797. doi: 10.3390/antibiotics15080797 (PMC13509231; doi:10.3390/antibiotics15080797)
Supplement: Supplementary file 1 [file antibiotics-15-00797-s001.zip › antibiotics-4365861-supplementary/Supplementary Materials S2.pdf]

Therapy of diabetic foot osteomyelitis

What influence do the following factors have in the decision-making process to treat diabetic foot osteomyelitis primary conservativelyto directly perform bone/extremity preserving surgery (debridement without resection, Ilizarov, etc.) or to directly perform an internal bone resection or amputation? For each category, please indicate whether this is more a factor for primary conservative or primary surgical treatment (limb preservation vs. amputation), or whether this factor has no influence on the therapy decision.

Only one X can be set per question.

1) *Name (optional)* ______________________________

2) *Medical specialty* ______________________________

3) *Hospital/ Employment* ______________________________

4) *I treat patients with diabetic foot osteomyelitis* ☐ Yes ☐ No

Patient-specific & environment-specific factors	***No influence***	***Conservative***	***Bone-/Limb sparing surgery***	***Bone resection/Amputation***
5) Local ischemia	☐	☐	☐	☐
6) Limb ischemia	☐	☐	☐	☐
7) Peripheral neuropathy	☐	☐	☐	☐
8) Renal failure & chronic kidney disease	☐	☐	☐	☐
9) Previous amputation	☐	☐	☐	☐
10) Multimorbid patient	☐	☐	☐	☐
11) Long history of non-healing ulcer (> 6 months)	☐	☐	☐	☐
12) Non-compliant patient	☐	☐	☐	☐
13) Absence of a treatment network: No internist/infectiologist available	☐	☐	☐	☐
14) Absence of a treatment network: No surgeon available	☐	☐	☐	☐
15) Absence of a treatment network: Lack of required follow-up (e.g. stump conditioning, Offloading)	☐	☐	☐	☐

**Osteomyelitis-specific factors**	***No influence***	***Conservative***	***Bone-/Limb sparing surgery***	***Bone resection/Amputation***
16) Exposed bone	☐	☐	☐	☐
17) Probe-to-bone (PTB) test positive	☐	☐	☐	☐
18) Localisation: Forefoot	☐	☐	☐	☐
19) Localisation: Heel	☐	☐	☐	☐
20) Extensive bone destruction (x-ray)	☐	☐	☐	☐
21) Biomechanical disturbances (e.g. charcot foot, rocker bottom foot)	☐	☐	☐	☐

Concomitant wound-specific factors	***No influence***	***Conservative***	***Bone-/Limb sparing surgery***	***Bone resection/Amputation***
22) Abscess	☐	☐	☐	☐
23) Erysipelas/ Concomitant progredient soft tissue infection	☐	☐	☐	☐
24) Necrosis	☐	☐	☐	☐
25) ↑ Ulcer size/ wound grade	☐	☐	☐	☐
26) Gangrene	☐	☐	☐	☐
27) Large/Progredient soft tissue loss	☐	☐	☐	☐
28) Not coverable soft tissue loss	☐	☐	☐	☐
29) Sepsis sensu strictu	☐	☐	☐	☐

30)	*Does the patient's wish have an influence on the therapy decision? (Conservative treatment vs. limb-sparing surgery vs. amputation)*	☐ Yes<br>☐ No
31)	*Does the wish of the relatives have an influence on the therapy decision? (Conservative treatment vs. limb-sparing surgery vs. amputation)*	☐ Yes<br>☐ No
32)	*Does the referrer's wish have an influence on the treatment decision? (Conservative treatment vs. limb-sparing surgery vs. amputation)*	☐ Yes<br>☐ No
33)	*What else is an important factor determining a conservative or a surgical approach (limb-sparing vs. amputation) to treat diabetic foot osteomyelitis?*	______________________________
